# Larval Survival of Fuller's Rose Weevil, *Naupactus cervinus*, on Common Groundcover Species in Orchards of New Zealand Kiwifruit

**DOI:** 10.1673/031.008.5101

**Published:** 2008-09-10

**Authors:** David P. Logan, Bridget J. Maher, Shirley S. Dobson, Patrick G. Connolly

**Affiliations:** ^1^ The Horticulture and Food Research Institute of New Zealand Ltd. (HortResearch), 412 No. 1 Road, RD2, Te Puke, New Zealand; ^2^ HortResearch, Private Bag 92 169, Auckland, New Zealand

**Keywords:** *Actinidia* spp. Fuller rose beetle, larvae, polyphagy, Lolium perenne, *Trifolium repens*, *Ranunculus repens*, *Duchesnea indica*, *Rumex obtusifolius*

## Abstract

Fuller's rose weevil, *Naupactus cervinus* (Boheman) (Curculionidae: Entiminae), is an important quarantine pest of New Zealand kiwifruit exported to Asian markets. Both adults and larvae are considered to be polyphagous. In this study, the survival of larval *N. cervinus* was estimated on common groundcover species of kiwifruit (*Actinidia* spp.) in the Bay of Plenty, the main region in New Zealand where kiwifruit is grown. The botanical composition of groundcover in commercial kiwifruit orchards, characterised by survey, was dominated by ryegrass (*Lolium perenne*), with white clover (*Trifolium repens*), creeping buttercup (*Ranunculus repens*), wild strawberry (*Duchesnea indica*) and broadleaf dock (*Rumex obtusifolius*) in lower abundance. Survival to mature larvae or adult was relatively low (·11%) for *N. cervinus* introduced as neonates to field plots or potted ryegrass, white clover and broadleaf dock. White clover was a more favourable host for survival to adults than ryegrass. This study suggests that increased survival of *N. cervinus* larvae may occur where white clover and large dock plants are abundant, but that survival is likely to be highly variable because of the heterogeneous availability of preferred host plants and host plant quality. These data suggest that larval polyphagy is a strategy that enables *N. cervinus* to persist at low densities in kiwifruit orchards despite variation in the quality and diversity of groundcover.

## Introduction

*Naupactus cervinus* (Boheman) (Curculionidae: Entiminae), is a flightless weevil from Argentina that has become cosmopolitan in distribution (Chadwick 1965a; AlonsoZarazaga and Lyal 1999). It has a mixed taxonomic history with many junior synonyms including *Pantomorus cervinus* (Boheman), *P. godmanni* Crotch, *Asynonychus cervinus* Crotch, *A. godmanni* Crotch and *Aramigus julleri* Horn (Chadwick 1965b; Alonso-Zarazaga and Lyal 1999). The accepted common name in the USA is Fuller rose beetle (Bosik 1997); in New Zealand and Australia, it is known as Fuller's rose weevil (Penman 1984; Carne 1987). Larvae of *N. cervinus* are regarded as a minor pest of citrus in Australia, Brazil, and Florida because of root herbivory (Hely 1948; Tarrant and McCoy 1989; Lanteri et al. 2002). In New Zealand kiwifruit, root herbivory by larvae and leaf herbivory by adults are not regarded as problems. However, eggs laid on fruit are, or have been, a quarantine barrier for New Zealand exports of kiwifruit to Japan, South Korea and some smaller markets (Steven 2005). Eggs on fruit have also been a quarantine barrier for exports of citrus from California and Australia to markets in East Asia (Lakin and Morse 1989; Madge et al. 1992; Anon. 2004).

In Argentina, both males and females occur but populations established elsewhere consist only of females that reproduce by parthenogenesis (Lanteri and Normark 1995). In Australia, New Zealand and California, *N. cervinus* has a one-year life-cycle with adults emerging from pupation sites in soil between mid summer and early autumn (Hely 1948; May 1979; Morse et al. 1988). Adults are flightless and their dispersal may largely be due to human agency. Longevity is typically 3–6 months during which between 100 and 1000 eggs are laid in batches of about 20–30 eggs. Longer-lived weevils lay most eggs (Chadwick 1965a; Masaki et al. 1996; Masaki and Kadoi 1997). Crevices in a wide variety of artificial and natural substrates are used as oviposition sites. Newly-hatched larvae burrow into soil and feed externally on roots until development is complete some 6–9 months later (Masaki and Kadoi 1997; Masaki and Nakahara 2000).

Both adult and larval *N. cervinus* are polyphagous, having been recorded feeding on a wide variety of cultivated plants and weeds (Chadwick 1965a; Masaki and Kadoi 1997). Not all hosts are likely to be equally preferred or of equal value for growth and survival. Polyphagous insects tend to have a hierarchy of preference (e.g. Firempong and Zalucki 1990; Thompson 1998), and sometimes have a narrower host range at the individual level than at the species level because of intrinsic genetic diversity and heterogeneous availability and quality of hosts (Howard et al. 1994; Walter 2003). In New Zealand, Maher and Logan (2004) found that adults of *N. cervinus* preferred to feed on the orchard groundcover plants white clover (*Trifolium repens*) and broadleaf dock (*Rumex obtusifolius*) rather than kiwifruit (*Actinidia deliciosa*) and citrus. Further, when given a choice of ryegrass (*Lolium perenne*) and white clover, adult *N. cervinus* aggregated strongly in the latter (Logan and Maher, unpublished observations). The host plant has a strong influence on adult longevity and fecundity (Masaki and Kadoi 1997; Maher and Logan 2004) and as a result, host plant availability is likely to be important in the population dynamics of *N. cervinus*. Host preference and performance of larvae on different hosts is less well studied than for adults. Masaki and Takahashi (1999) reared larvae of *N. cervinus* in the laboratory on potatoes in soil, and on potted strawberry and citrus. Survival after 90 days at 24°C was relatively high:64% on strawberry, 66% on citrus and 92% on potato. There were significant differences in body weight after 90 days, with only 16% of larvae reared on strawberry becoming fully-grown compared with all larvae reared on potato. Survival of larvae in the field, including in New Zealand kiwifruit orchards, has not been measured. Here the survival of larval *N. cervinus* was estimated on common groundcover species of kiwifruit grown in the Bay of Plenty, the main region of kiwifruit production in New Zealand. While roots of kiwifruit vines may also support *N. cervinus* larvae, we focussed on groundcover because it is potentially more amenable to manipulation. The botanical composition of groundcover in kiwifruit orchards was also characterised by survey. These data provide a basis for understanding the population dynamics of *N. cervinus* in New Zealand kiwifruit orchards.

## Materials and Methods

### Recovery of seeded Fuller's rose weevil in field plots

To estimate survival of *N. cervinus* in groundcover, plots (0.5 × 0.5 m square) were established between two kiwifruit blocks at the HortResearch kiwifruit orchard at Te Puke, Bay of Plenty. The sides of each plot were enclosed by black polythene sheeting to a depth of 200 mm. Plots had a mixed groundcover of grasses (*Lolium perenne*, *Poa annua*, *Holcus mollis*, *Digitaria sanguinalis*), white clover (*Trifolium repens*), and some broadleaf weeds (*Cerastium glomeratum*, *Rumex obtusifolius*, *Veronica persica*). Newly hatched larvae (n = 150) of *N. cervinus* were added to the surface of 36 plots during the autumn (22–26 April) of 2002. Based on limited data from commercial kiwifruit orchards, this density of larvae may be ten times higher than is typical and was selected to increase the chance of recovery. Larvae were from eggs laid by a colony of at least 200 adults collected from five geographically separated commercial kiwifruit orchards. Adults were fed on citrus leaves and laid eggs in folds of waxpaper or plastic sheets, and were assumed to be typical of *N. cervinus* in the Bay of Plenty. This is likely to be a fair assumption as, in New Zealand, *N. cervinus* reproduce parthenogenetically and are relatively genetically uniform (Mander et al. 2003). Soil in six plots was excavated to a depth of 150 mm on 8 July (days 73–77 after larval introduction), 14 October (days 175–179), 13 November (days 201–205), 16 December (days 234–238), and 3 February (days 283–287). Larvae and pupae of *N. cervinus* were extracted from soil by wet-sieving and flotation in magnesium sulphate solution using a method similar to Kain and Atkinson (1976). Subsamples of larvae were confirmed as *N. cervinus* based on the following combination of taxonomic characters: a mainly colourless head that is retracted into the prothorax, a length of no more than 10 mm, and with postdorsal seta I twice as long as postdorsal seta II on abdominal segments II–IV (May 1977). Number of larvae and pupae, and dry weight of roots were recorded for three depths of soil, 0–50 mm, 50–100 mm and 100–150 mm for each sample. Dry root mass was determined after drying roots retrieved during wet-sieving, at 60°C for 96 h. Cages made of wood and screen mesh (1 mm aperture) in the form of a pyramid with catch container at the apex, were placed over each plot on 15 November 2002 and checked weekly to remove emerged adults until 22 April 2003.

Data for numbers of larvae recovered were analysed with a binomial Generalized Linear Model. The number of days since introduction of larvae (Days) was considered an ordered factor to see if Days had an effect on the proportion of larvae found at the various levels. The model assumed the conditions affecting survival of larvae were identical for each plot and that the number of larvae burrowing deeper than 150 mm could be ignored, either because it was zero, or if not zero, was a similar proportion in all plots. Natural mortality after days 175–179 was assumed to be negligible also, as was the number reaching maturity. Equality of larval numbers found at each depth was tested by comparison with the χ2 distribution. The number of larvae recovered at each depth was compared with root mass at the same depth by correlation.

### Survival on potted plants

Three experiments were undertaken to determine whether initial larval density affected survival to mature larva or adult stage. In experiment 1, dock (*R. obtusifolius*) plants were dug from the groundcover of the HortResearch kiwifruit orchard at Te Puke, washed to remove soil and invertebrates, and established in 5-L pots with screened Te Puke sandy-loam soil. Different numbers (10, 40, 70, 100) of newly hatched larvae were added to each of 10 pots on 24 May 2002. In experiment 2, newly hatched larvae were added to 32 pots at either 10 (16 pots) or 100 larvae (16 pots) per pot on 7 August 2002. All pots had been filled with screened Te Puke sandyloam soil and sown with a commercial lawn seed mix (80% *L perenne*, 20% *T. repens* and *Festuca* spp.). After inoculation with larvae, all pots in experiments 1 and 2 were kept on a bench in a glasshouse. Fine mesh covers were added to the top of all pots in experiments 1 and 2 in December 2002 and emerged adults were collected once each week between 23 January and 11 April 2003, after which no further adults were found. In experiment 3, newly hatched larvae were added to 36 pots on 4–8 July 2003. Pots had been filled with screened soil and planted with a one-year-old dock plant grown from seed, Endophyte-free ryegrass (*L. perenne*) or white clover (*T. repens* ‘Kopu II’). Sowing rate for the latter two hosts was approximately 50 seeds/pot, which is about five times the commercial rate of 10 kg/ha for ryegrass and 5 kg/ha for white clover. Both white clover and ryegrass were sown approximately two months prior to larval introduction. Half of each group of 36 pots was inoculated with 40 and half with 100 larvae per pot so that there were 18 pots for each host × density grouping. Potted plants were grown in a greenhouse and initially watered daily until September when they were moved to weed matting in open air and watered once aweek. Watering was by automatic overhead sprinkler and lasted for 30 minutes. Soil in the pots was hand-sorted in early February 2004 to recover and count numbers of mature larvae and pupae.

After experiments to identify whether initial density of larvae influenced survival, the effect of host plant on survival to full-grown larvae or pupae was tested for white clover, ryegrass and a 50:50 mix of white clover and ryegrass (experiment 4). Air-dried Te Puke sandy-loam was sieved through 1-mm mesh and added to 180 2-L pots; 60 pots were planted with ryegrass, white clover or ryegrass + white clover at a rate of approximately 50 seeds per pot. Newly hatched larvae (n = 40 per pot) were introduced to the soil surface of each pot on 21 July 2004 when plants were three months old. Pots were buried in a field plot to ground level so that larvae experienced field temperatures. Six pots of each host plant were removed from soil on September 22–24 (63–65 days after larvae were introduced) and November 17–19 (119–121 days), eight plants per host on December 22–23 (154–155 days), and ten plants per host on February 4 and 7 (198, 201 days) and surviving larvae counted after wet-sieving (sieve aperture sizes: 212 µm, 1 mm and 2.5 mm) in a solution of magnesium sulphate at 1.05 SG. Cages were added to the top of each remaining pot (30 per host) and any emerged adults were removed weekly between February 1 and May 30. The numbers of larvae surviving under the different circumstances were analysed using the GLM functionality in R (R Development Core Team 2005). In most cases, the data were highly underdispersed because of a large number of zeros; in some cases it was overdispersed. To allow for the dispersion, the “quasibinomial” GLM family was considered more appropriate than the standard binomial family, which is assumed to have a dispersion parameter of 1.

The head-capsule width of newly hatched larvae (n = 30) and of larvae recovered from pots (n = 30, or all recovered survivors) was measured with the aid of an eyepiece graticule on a dissecting microscope, to track larval growth. Hourly temperature was measured at 10 mm depth in soil adjacent to pots by temperature logger from 29 July 2004 until no further adults had emerged in May 2005. Dry weight of root mass recovered after sieving was determined by drying roots in an oven at 80°C for 48 hours. Dry root mass for the three plant hosts (white clover, ryegrass, ryegrass + white clover) and for pots for each of four sampling dates was analysed using an unbalanced two-way ANOVA after transformation by square- root to reduce variance heterogeneity. Plots were examined to ensure that residuals were normally distributed and evenly spread across groups. Significance was at *P* <0.05 and analysis was carried out in Genstat 9.1 (Genstat 2006).

### Groundcover survey

The groundcover of 100 commercial blocks, 50 each of the two dominant commercial kiwifruit cultivars ‘Hayward’ and ‘Hort16A’, was sampled to determine the most abundant groundcover species. Blocks were distributed throughout the area of kiwifruit production in the western Bay of Plenty, an area approximately 100 km long and extending approximately 20 km from the coastline. The composition of the groundcover was determined by basal-hit, single-point sampling (Owensby 1973) for 50 points within each block between mid February and late March 2004. Most kiwifruit in New Zealand is grown using a pergola support system enabling the groundcover to be sampled under the entire canopy. Each point was located using a list of random numbers for the number of steps between samples. Direction between samples was alternated by 90° in a zig-zag pattern. Roy et al. (1998) and Taylor (1980) were used as references for plant identification. Diversity of groundcover for each cultivar was summarised by calculating α of the log series and the Berger-Parker index for each site and comparing these values by two-sample t-test. The log series is a distribution model for species abundance described by the parameter α and the total number of individuals, N (Magurran 1988). The Berger-Parker index (d) is a measure of species dominance and was calculated as d = N_max_/N where N_max_ is the number of the most abundant species (Magurran 1988).

**Table 1.**
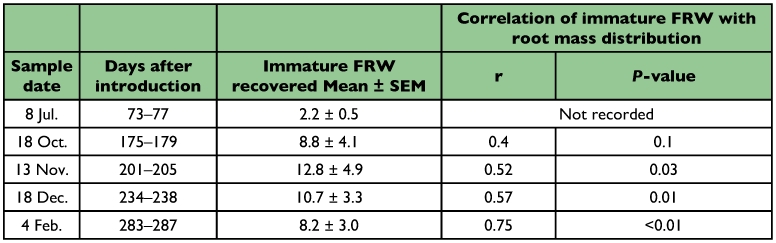
Recovery of larvae and pupae of Fuller's rose weevil (FRW) from 0.25 m^2^ plots and correlation of distribution at three depths 0–50, 51–100 and 101–150 mm between root mass and soil-dwelling immatures. 150 newly-hatched larvae were introduced to each plot.

## Results

### Recovery of seeded Fuller's rose weevil in field plots

Relatively few larvae were recovered on each sampling date and very few adults were found in traps (mean per plot ± sample SD: 2.3 ± 2.3) (Table 1). Pupae (mean per plot ± SD: 2.7 ± 2.3) were found in the top soil stratum (0–50 mm) of samples on days 283–287. Days after introduction of larvae to field plots had no effect on the number of larvae recovered (*P* > 0.05 in every measure) or on the proportion of larvae at each of the three soil depths (*P* > 0.05). From that observation, steady state conditions were assumed to have formed so that the number of larvae found at each depth reflected a preference for that depth. The number of larvae combined for all sampling dates differed at the three depths (χ^2^ = 47.5, df = 30, *P* = 0.02). More larvae occurred at 0–50 mm deep (n = 181) than at 51–100 mm (n = 52) and 101–150 mm (n = 10). Number of larvae and root mass at each depth were positively correlated for the final three sampling periods (Table 1).

### Survival on potted plants

In experiments 1 and 2, *N. cervinus* completed development from first instar to adult on broadleaf dock and on grass, although survival was low (Table 2). There was no effect of density on survival of larvae reared on dock (*P* > 0.05) (Table 2). There was some evidence that an initial high density of larvae (100/pot) affected survival on grass (*P* = 0.02), although the result must be treated with caution as there were only two pots with surviving adults in the low density treatment (10/pot). The difference may reflect the precision of the measurements more than any difference between the densities. Adults emerged slightly earlier from pots with dock (week of 50% emergence = 7–14 February) than from pots with grass (28 February-7 March), reflecting the earlier date of introduction to the former. In experiment 3, density had no significant effect on the number of larvae recovered, nor was there any interaction between density and host (*P* > 0.05). There was a trend for survival on ryegrass to be lower than for white clover (*P* ∼ 0.06). Neither could be compared in a reliable way to dock, on which mortality was almost 100% at both densities.

**Figure 1.**
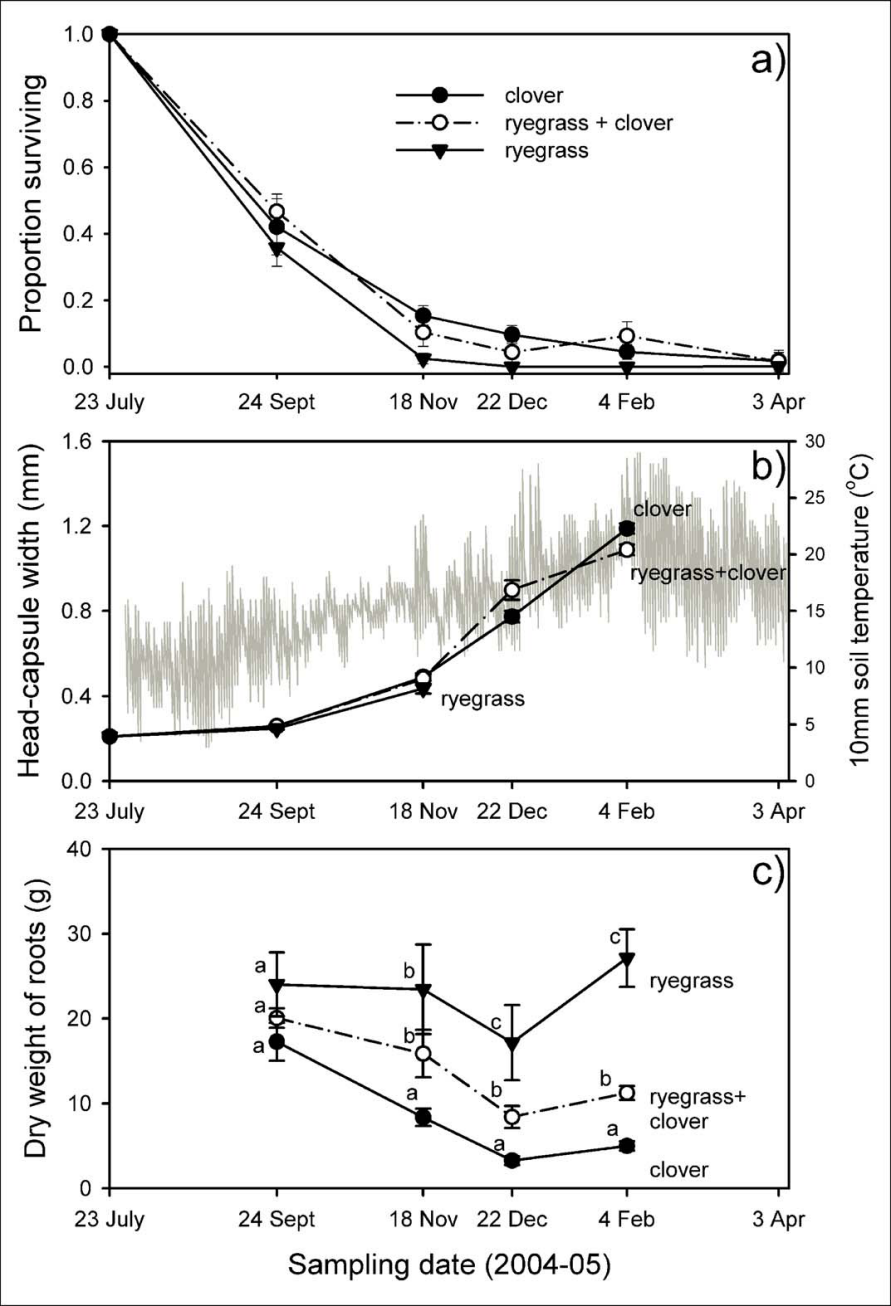
a) Proportion of larvae (24 September-4 February) retrieved from pots and adults (3 April = median date of emergence) trapped after emerging, b) Change in mean head-capsule width of larvae with time (lines with symbols) and daily maximum and minimum soil temperature at 10 mm deep in bare soil adjacent to pots (grey line), c) Change in dry weight of roots within pots for the three diets. Mean values that share the same letter are not significantly different for the same sampling date. Error bars are one standard error of the mean. Symbols and line formatting is consistent for the three diets (clover, ryegrass + clover, ryegrass) for all three graphs.

In experiment 4, there was an interaction between the effect of host and the sampling date, so that the differences between the hosts were strongly influenced by the time of year in which they were compared (Figure 1a). Consequently, the effect of hosts was calculated separately for each sampling date. No differences were significant for samples on 22–24 September (63–65 days after introduction) (*P* > 0.05). Numbers of larvae were significantly lower in ryegrass than in clover and ryegrass + clover on 17–19 November (119–121 days) (*P* = 0.02). For samples taken on 22–23 December (154–155 days), numbers of larvae in clover were not different from those in ryegrass + clover (*P* > 0.05), but neither could be compared with ryegrass, on which there were no survivors. Similarly, on 4–7 February (198–201 days), numbers of larvae in clover were not different from those in ryegrass + clover (*P* > 0.05) but neither could be compared with ryegrass, on which there were no survivors. The same number of adults emerged from pots with white clover (mean ± SEM: 0.7 ± 0.2) and ryegrass + clover (0.7 ± 0.2) (*P* > 0.05). Numbers of survivors from both treatments were different to those from ryegrass (0.03 ± 0.03) (P < 0.03).

Mean dry root mass differed for hosts (F = 53.0; df = 2,79; *P* < 0.001) and sampling date (F = 13.3; df = 3,79; *P* < 0.001). Differences between hosts were influenced by time of the year (F = 2.8; df = 6,79; *P* = 0.015) and hosts were compared for each sampling date by Fisher's LSD test at α< 0.05. No differences in dry root mass occurred on 22–24 September. Clover root mass differed from the other two hosts on the second sample date, 17–19 November, and all three hosts differed in root mass dry weight for the final two sampling dates in December and February (Figure 1c).

**Table 2.**
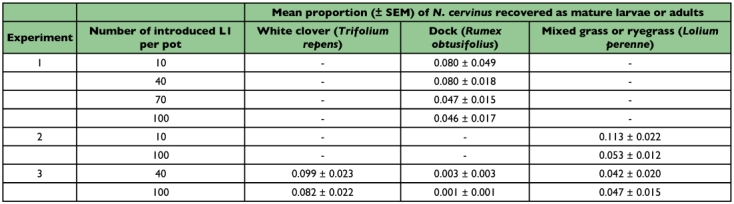
Effect of first instar (L1) density on survival to adults (experiments 1, 2) or mature larvae (experiment 3) of Fuller's rose weevil (Naupactus cervinus) for selected potted groundcover hosts.

### Groundcover survey

**Figure 2.**
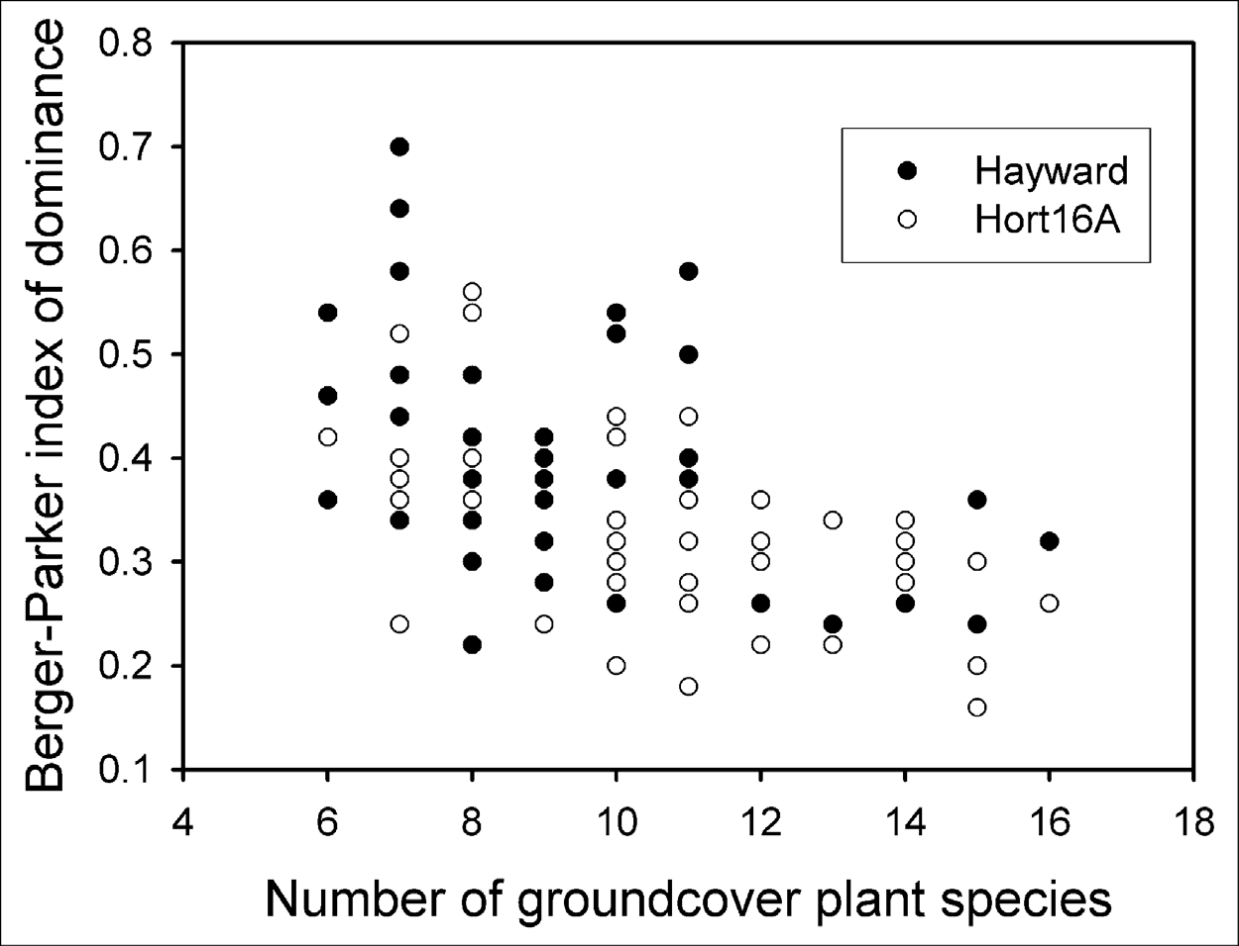
Berger-Parker index of dominance and number of groundcover species for 50 blocks of the kiwifruit cultivars ‘Hayward’ (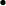) and ‘Hort16A’ (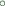) in the Bay of Plenty, New Zealand. The Berger-Parker index (d) differs between cultivars (P < 0.05). Values of d approaching 1 indicate increasing dominance by the most common species; when d = 1, groundcover would consist entirely of a single species.

A total of 53 groundcover plant species were identified in the survey of ‘Hayward’ and ‘Hort16A’ kiwifruit blocks; 39 species were common to both cultivars. The most common groundcover species for both kiwifruit cultivars and the only species present at all sites was ryegrass (*L. perenne*) (mean incidence ± sample SD: ‘Hayward’, 29.3 ± 10.8%; ‘Hort16A’, 23.6 ± 9.9%). Bare ground made up 22.0 ± 19.0% and 21.0 ± 14.8% of groundcover of ‘Hayward’ and ‘Hort16A’ kiwifruit blocks, respectively. A further 30% of groundcover of both cultivars was made up by white clover, creeping buttercup (*Ranunculus repens*), wild strawberry (*Duchesnea indica*) and broadleaf dock, in order of abundance. Groundcover plant diversities as indicated by α of the log series were similar for ‘Hayward’ and ‘Hort16A’ (*P* > 0.05), but dominance by the most common plant species differed between groundcovers of each cultivar (*P* = 0.007) (Figure 2). Ryegrass was the most abundant groundcover species in 44/50 ‘Hayward’ sites and 33/50 ‘Hort16A’ sites.

## Discussion

Survival to mature larvae or adults for *N. cervinus* introduced as neonates to field plots and potted groundcover plants was, in general, highly positively skewed; some pots or plots had relatively high larval survival but many had very low survival or no surviving larvae. This survival pattern implies that the density of neonate larvae, in the range tested, may not have a large influence on establishment and that other factors were more important. Average survival was in the range 4–11%, with two exceptions. Survival of neonates introduced to broadleaf dock in experiment 1 was 5–10% as expected, but low in experiment 3 (<1%). Similarly, survival to adults or mature larvae on ryegrass or mixed grass was 4–11% in experiments 2 and 3, but was <1% for experiment 4. Dock plants removed from the field and replanted in experiment 1 were mature plants with many large tap roots. In contrast, the dock plants used in experiment 3 were smaller with few large tap roots, and hence may have provided less opportunity for larval feeding. Grass appeared to be much more uniform in pots and differences in survival are difficult to explain. Small plant size for docks may have affected weevil survival and led to an unfair comparison with ryegrass and white clover in experiment 3. Herbivory by *N. cervinus* larvae was associated with a reduction in root mass of white clover and ryegrass in experiment 4 between spring and early summer. Ryegrass began to recover by mid-summer and this probably reflected both the significant decline in numbers of *N. cervinus* larvae and the warmer temperatures. Root growth of white clover recovered more slowly than ryegrass and there is potential for the white clover component of groundcover to be reduced by *N. cervinus* herbivory.

Ryegrass was the dominant groundcover species and is likely to be a common host plant of *N. cervinus* larvae in kiwifruit orchards in the Bay of Plenty. Although *N. cervinus* can complete development on ryegrass, white clover may be superior as a host. Despite having significantly higher root mass in summer, fewer adults emerged from pots with ryegrass than with white clover, either alone or in combination with ryegrass. Similarly, Hardwick et al. (1999) found that white clover was preferred to ryegrass by larvae of the closely related white-fringed weevil, *Naupactus leucoloma* (Boheman). Populations of *N. leucoloma* reached their highest levels in pasture dominated by legumes such as white clover (King and East 1978). Legumes are also preferred hosts for other broadnosed (entimine) weevils such as *Hypera postica* (Gyllenhal), *Sitona lepidus* Gyllenhal, and *S. discoideus* Gyllenhal (Latheef et al. 1979; Bright 1994). Performance of larval *N. cervinus* on white clover is consistent with the preferences and increased fecundity of adult *N. cervinus* on white clover compared with other potential hosts in New Zealand kiwifruit (Maher and Logan 2004). Clover-dominated groundcover may thus favour population growth by *N. cervinus* in orchards. In addition to groundcover, *N. cervinus* may also feed and complete development on kiwifruit roots. Larvae of the closely related weevils *N. xanthographus* (Germar) and *N. leucoloma*, and the polyphagous garden weevil, *Phlyctinus callosus* Boheman, are able to complete development on kiwifruit (*Actinidia chinensis*) roots (Ripa 1986; D. Logan, unpubl.). For *N. Xanthographus*, other fruit crops such as citrus are superior hosts to kiwifruit (Ripa 1986). In this study, larvae were predominantly found in the top 100 mm of the soil surface, where roots of ground cover plants are most abundant.

Relatively low larval survival in this study is consistent with the observation of low adult densities in kiwifruit orchards in the Bay of Plenty. Commercial kiwifruit orchards identified by packhouse operators in the kiwifruit industry as having ‘high’ populations of *N. cervinus* had average annual catches in emergence traps of 2–3 adults m (C. McKenna et al., unpublished observations). By comparison, in New Zealand pasture or lucerne crops, populations of some other broad-nosed weevils such as *S. lepidus*, *S. discoideus* and *N. leucoloma* can exceed >30 late instar larvae or pupae m^-2^ (Wightman 1986; Hardwick and Prestidge 1994; Brownbridge et al. 2006). Low larval survival in this study contrasts with that of Masaki and Takahashi (1999). They reared *N. cervinus* on potatoes and on potted strawberry and citrus, and recorded larval survival of more than 60% to late instar. Environmental conditions and hosts differed between studies. Masaki and Takahashi (1999) reared larvae in the laboratory at 24°C; in this study, larvae were reared under field or greenhouse conditions and exposed to more variable and in general cooler temperatures. Chadwick (1965) considered that most mortality in *N. cervinus* occurred as pupae and newly-emerged adults. Mortality in this study occurred mainly in the early instars, with numbers of older larvae reaching a near steady state. Factors limiting establishment in the field could include failure to find adequate food, very dry or wet periods, extremes of temperature, and predation by natural enemies. These factors, or a combination of these factors may have been an important larval mortality in this study. This is partly implied by relatively high survival (>60% to mature larvae) of *N. cervinus* on potatoes in the laboratory (Masaki and Takahashi 1999) and on artificial diet (30–50% to adults)(G. Clare, HortResearch, Auckland, personal communication). Dry soil reduced establishment of black vine weevil larvae, *Otiorhynchus sulcatus* (Fabricius) (Shanks and Finnigan 1973), but was probably not a factor in this study as soil was kept relatively moist by irrigation or rainfall after neonates were introduced. Conversely, poorly drained soil may be detrimental to establishment by young larvae; neonate *N. cervinus* introduced to artificial diet for rearing were trapped and drowned by excessive moisture (G. Clare, personal communication). The pest status of *N. cervinus* on citrus, typically grown commercially on sandy free-draining soils, may indicate a preference for relatively dry soil conditions and for soils with a relatively high fraction of sand. Soil in pots was a sandy loam that had been dried and sieved to reduce poor drainage and its negative impact on larval establishment. Extremes of soil temperature were probably not a significant mortality factor, particularly in the mild temperate climate of the Bay of Plenty, as neonates of *N. cervinus* tolerate a wide range of temperatures (Tarrant and McCoy 1989; Masaki et al. 1996). The latitudinal spread of *N. cervinus* from Nelson, New Zealand (41°17′S) to Ottawa, Canada (45°22′N) (Chadwick 1965) also suggests a wide tolerance to temperature differences. Host-finding behaviour in soil typically relies on host volatiles and is probably impeded when soil is compacted such as occurs in orchard interrows (e.g. Vogeler et al. 2006). Soil with bulk densities of 1.1 g cm and a preponderance of small diameter pores (0.15 mm) restricted movement by first-instar corn rootworms *Diabrotica virgifera virgifera* (Gustin and Schumacher 1989). First instar *N. cervinus* have a similar head-capsule width as first-instar corn rootworm (∼0.2 mm) and may be similarly affected by compacted orchard soils. Hamon et al. (1990) suggested that carabids could reduce numbers of larvae of *Sitona lineatus* (L.) by up to 10% in field beans. No carabids were observed in soil in pots. The ants, *Pheidole rugulosa* and *Paratrechina* sp. established nests in 10% of pots in experiment 4 and the adventive predatory staphylinid *Thyreocephalus* sp. was also observed in field plots and some pots. The latter may have been responsible for some larval mortality; however, the presence of an ant colony was not clearly associated with an absence or low number of larvae in pots. Naturally-occurring entomopathogenic fungi and nematodes infect and kill the soildwelling larvae and pupae of *N. cervinus* and other weevil species (Beavers et al. 1983; Smith et al. 1997). However few cadavers were recovered from pots and no attempt was made to isolate pathogens or to rear larvae to identify infections.

The high level of larval mortality is likely to be a key factor governing population size in *N. cervinus*. In New Zealand at least, *N. cervinus* populations appear to be low in comparison with those of some other entimine pest weevils in pasture. According to Bloem et al. (2002), adult *N. cervinus* may be abundant at particular sites and times of the year in Florida, but they provided no data. In citrus-growing areas in southern Australia, weekly counts of *N. cervinus* in citrus canopies were always less than 10 per tree (Madge et al. 1992), a density probably not significantly higher than found in Bay of Plenty kiwifruit. Observations that adult *N. cervinus* can be abundant in pre-production kiwifruit orchards where mowing is infrequent suggest that density of groundcover is important. In general, however, there are inadequate population data to draw robust conclusions about favourable environments for *N. cervinus.* This study suggests that increased survival of *N. cervinus* larvae may occur where white clover and large dock plants are abundant, but that survival is likely to be highly variable because of the heterogeneous availability of preferred host plants and host plant quality. We suggest that larval polyphagy is a strategy that enables *N. cervinus* to persist at low densities in kiwifruit orchards despite variation in the quality and diversity of groundcover. Further studies of *N. cervinus* in New Zealand and elsewhere would be valuable in assessing the role of groundcover and tree hosts relative to other factors such as climate in determining population size.
